# Magnetism and 3D Electron Diffraction Solution of Hydrated Rubidium–Ruthenium Oxide Rb_2_Ru_2_O_7_·H_2_O

**DOI:** 10.1021/acs.inorgchem.6c00345

**Published:** 2026-02-18

**Authors:** Krystof Chrappova, Jeremiah P. Tidey, Christopher Bell, Simon R. Hall

**Affiliations:** † School of Chemistry, 1980 University of Bristol, Cantock’s Close, Bristol BS8 1TS, U.K.; ‡ Department of Physics, 2707 University of Warwick, Coventry CV4 7AL, U.K.; ¶ School of Physics, University of Bristol, Tyndall Avenue, Bristol BS8 1TL, U.K.

## Abstract

The crystal structure of Rb_2_Ru_2_O_7_·H_2_O was determined by three-dimensional electron diffraction from the individual crystallites of a solid-state powder product. Rb_2_Ru_2_O_7_·H_2_O crystallizes in space group *C*2/*c* (*a* = 7.841(3) Å, *b* = 12.500(3) Å, *c* = 8.392(2) Å, β = 93.57(4)°, Z = 4). The structure contains infinite chains that run normal to the (101) plane and consist of alternating RuO_6_ octahedra and square-pyramidal RuO_5_ units connected via shared O–O edges. Magnetic properties were measured on the bulk powder, showing a diamagnetic baseline from 300 K to 60 K with a small Curie tail below 55 K. The magnetic moment, calculated from the 1.8 K isotherm, saturates at *M* = 4.4 × 10^–3^ μ_B_ Ru^–1^, much less than would be expected for *S* = 1 ruthenium. Bond-valence-sum analysis indicates high-valent Ru, and the near-diamagnetic response is consistent with the edge-sharing Ru–Ru motif, where weak direct Ru–Ru overlap yields a local singlet.

## Graphical abstract

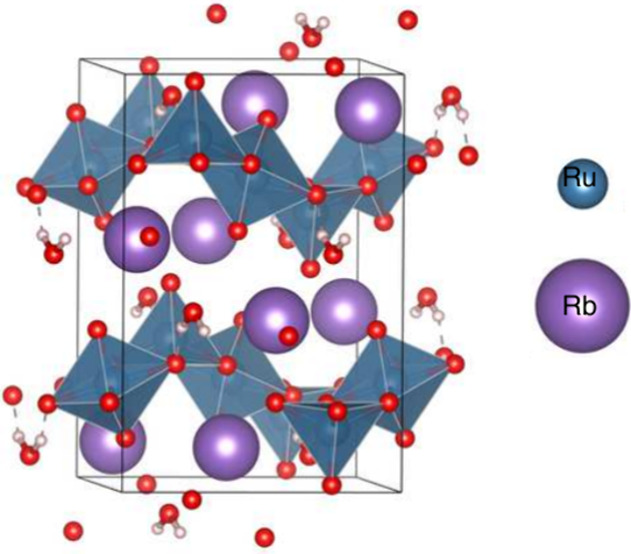

Ruthenium oxides display a wide range of unusual electronic ground states. Among alkaline metal ruthenates, SrRuO_3_ is an itinerant ferromagnet (*T*_Curie_ = 150 K), whereas its iso-structural analogue CaRuO_3_ remains paramagnetic when undoped.^1,2^ Sr_2_RuO_4_ exhibits unconventional superconductivity (superconducting *T*_c_ = 1.5 K).^3^

In the alkali-ruthenate family, intermediate valence Ru^4+/5+^ allow diverse extended frameworks. Two-dimensional honeycomb layers have been reported for Li_2_RuO_3_ and Na_2_RuO_3_, and isolated octahedral chains are present in Li_3_RuO_4_.^4–6^

Here, we present the previously unknown hydrated oxide Rb_2_Ru_2_O_7_·H_2_O, whose structure comprises isolated zigzag chains of alternating edge-sharing RuO_6_ octahedra and RuO_5_ square pyramids. The compound was solved by three-dimensional electron diffraction (3D ED) and its magnetic properties were determined on the bulk polycrystalline solid-state product.

A polycrystalline sample of Rb_2_Ru_2_O_7_·H_2_O was obtained by reacting ground Rb_2_CO_3_ (1.5 mmol) with RuO_2_ (0.75 mmol) in an alumina crucible at 1000 °C (4 h; 5 °C min^–1^ ramp). The structure (Figure 1) was solved from micrometer-sized crystals (Figure S1) using continuous rotation, selected-area 3D ED performed at 100(5) K. Rietveld refinement using the 3D ED model fits the laboratory powder X-ray diffraction (PXRD) pattern of the bulk sample well (Figure 2). Scanning electron microscopy with energy-dispersive X-ray spectroscopy (SEM/EDS) shows colocalization of both metals used in the synthesis (Figures S3 and S4). Transmission electron microscopy reveals the powder consists of polycrystalline, needle-like particles up to 6 μm in length, and the corresponding selected area electron diffraction shows rings that can be indexed to the lattice planes in the 3D ED structure (Figure S5, Table S3).

**Figure 1:**
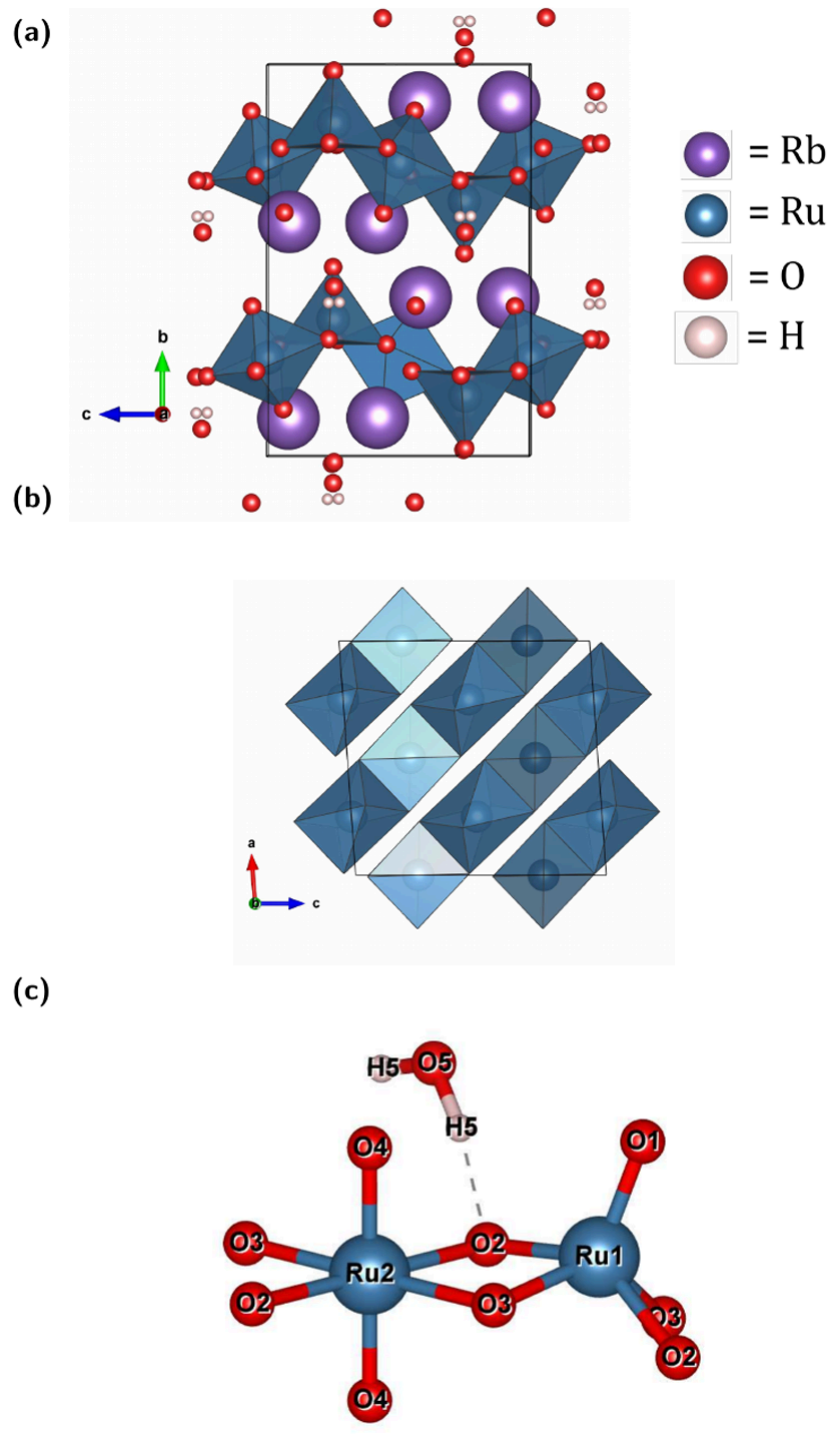
Polyhedral representation of (a) 3D ED structure solution projected onto (100) for Rb_2_Ru_2_O_7_·H_2_O in the monoclinic *C*2/*c* space group (*a* = 7.841(3) Å, *b* = 12.500(3) Å, *c* = 8.392(2), *Z* = 4, β = 93.57(4)°), (b) zig-zag chain of alternating RuO_6_/RuO_5_ projected onto (010). (c) Edge-sharing unit that comprises the nominal molecule labeled by crystallographically unique atoms.

**Figure 2:**
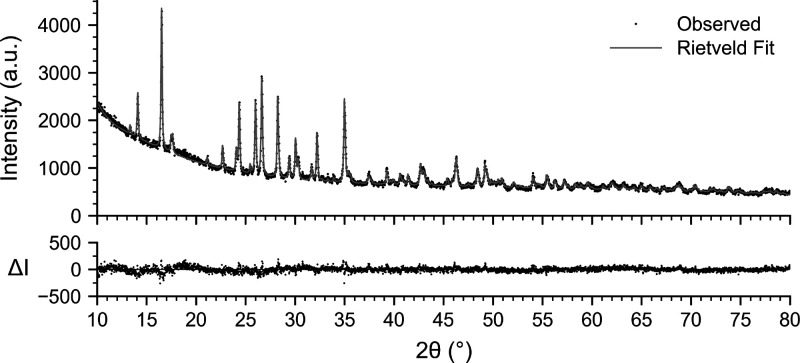
PXRD pattern of bulk Rb_2_Ru_2_O_7_·H_2_O with a Rietveld refinement using the structure solved by 3D ED (ICSD deposition: CSD 2514706) without internal standard. Rietveld refinement: χ^2^ = 1.8, *R*_wp_ = 4.5, *R*_exp_ = 3.4.

The results from 3D ED show two crystallographically independent Ru sites are present in the crystal structure (Figure 1c). Ru1 is five-coordinate, forming a distorted square pyramid with an apical Ru1–O1 bond of 1.656(8) Å and basal bonds Ru1–O3 = 1.896(6) Å (×2) and Ru1–O2 = 1.910(6) Å (×2). Ru2 is six-coordinate, with two short axial contacts Ru2–O4 = 1.741(6) Å and four equatorial contacts Ru2–O3 = 1.994(6) Å (×2) and Ru2–O2 = 2.032(5) Å (×2). The polyhedra share the O2–O3 edge, giving Ru1**···**Ru2 = 3.045(4) Å. Edge-sharing continues normal to the (101) plane (Figure 1).

Figure 3 (a) shows the temperature dependence of the molar susceptibility. A temperature-independent baseline χ_0_ = −1.06 × 10^–8^ m^3^ mol^–1^ persists from 300 to 60 K, followed by a Curie upturn below ∼55 K. A linearization of [χ­(*T*) – χ_0_]^−1^ against temperature over 5–55 K yields a Curie constant *C* = 1.32 × 10^–6^ m^3^ mol^–1^ K and an effective moment μ_eff_ = 0.65 μ_B_ Ru^–1^. The 1.8 K, 7 T isotherm saturates at *M* = 4.4 × 10^–3^ μ_B_ Ru^–1^, implying that only a small fraction of Ru sites carry *S* = ^1^/_2_ (or *S* = 1) moments, or that the moments form some kind of antiferromagnetic-like order whose antiparallel moments are not significantly canted at an applied field of 7 T. Rietveld refinement with an Al_2_O_3_ internal standard shows that 27% of the bulk mass is crystalline Rb_2_Ru_2_O_7_·H_2_O (Figure S2). All χ and *M* data are normalized to the crystalline Rb_2_Ru_2_O_7_·H_2_O mass fraction within the sample. Thus, trivial dilution by amorphous material has already been removed. Therefore, the vanishing net moment is intrinsic to Rb_2_Ru_2_O_7_·H_2_O.

**Figure 3:**
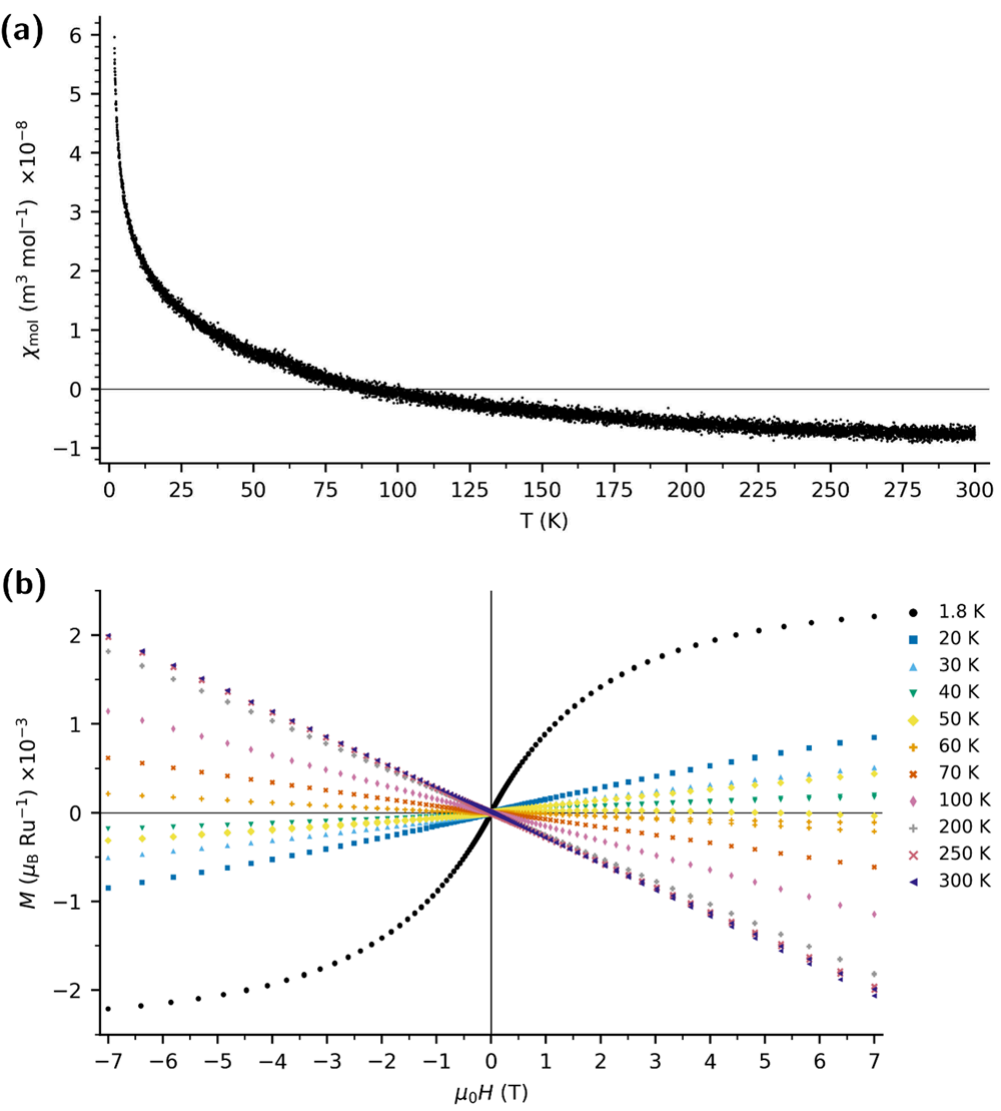
(a) Temperature dependence of the molar susceptibility, χ­(*T*), for bulk polycrystalline Rb_2_Ru_2_O_7_·H_2_O, zero field cooled with applied field of 1 mT. (b) Isothermal magnetization, *M*(μ_0_ *H*), at selected temperatures. All χ and *M* data are normalized to the crystalline Rb_2_Ru_2_O_7_·H_2_O mass fraction obtained by Rietveld refinement with an Al_2_O_3_ internal standard (*f*_cryst_ = 0.27) and values are reported per Ru in the crystalline phase.

Bond-valence-sum (BVS) analysis was carried out for both Ru coordination polyhedra. Using Brese–O’Keeffe parameters for Ru^4+^ (*R*_0_ = 1.834 Å, *B* = 0.37 Å) gives *V*_Ru1_ = 4.94 and *V*_Ru2_ = 5.04 v.u.^7^ Parameters for higher oxidation states are not available using this reference. Using the Gagné–Hawthorne Ru^4+^ parameters (*R*_0_ = 1.833 Å, *B* = 0.366 Å) produces similar results *V*_Ru1_ = 4.93 and *V*_Ru2_ = 5.02 v.u., whereas their Ru^5+^ set (*R*_0_ = 1.894 Å, *B* = 0.346 Å) increases the sum to *V*_Ru1_ = 5.89 and *V*_Ru2_ = 5.95 v.u.^8^ Parameters for Ru^6+^ are not available. Because the Gagné–Hawthorne parameter sets were calibrated predominantly for regular RuO_6_ octahedra, the distortion of the Ru2 octahedron and the short apical bond in Ru1 inflate the valence sum and the BVS is in this case semiquantitative allowing for both +5 and +6 formal charge.^9^

Both BVS-permitted oxidation states can plausibly produce Ru–Ru bonding combinations that yield a local singlet consistent with the observed near-diamagnetism. For Ru^5+^ (d^3^ + d^3^), edge-sharing Ru pairs permit three *t*_2*g*_–*t*_2*g*_ overlaps that form bonding combinations denoted σ, π, δ, as has been previously shown for the Li_2_RuO_3_ system.^10^ Six electrons can then fill this bonding set as σ^2^π^2^δ^2^, giving a closed-shell *S* = 0. For the Ru^6+^ (d^2^ + d^2^) analogue, either a molecular orbital configuration that results in *S* = 1 (σ^2^π^1^δ^1^) or closed shell configuration (σ^2^π^2^δ^0^) is possible. However, only the latter is consistent with the observed magnetism. The Ru1–Ru2 separation of 3.045(4) Å lies within the range where weak direct overlap has been invoked (viz., 2.76–3.06 Å in Y_5_Ru_2_O_12_).^11,12^

We conclude, therefore, that the magnetism here is likely governed through overlap of Ru–Ru neighbors. The open-shell Ru^6+^ molecular orbital configuration or Ru^4+^/Ru^6+^ disproportionation would contradict either the Curie constant or the single set of Ru–O distances and are thus ruled out.

## Supplementary Material



## Notes

Experimental procedures, details for the reported dynamical and SHELX-compatible kinematic 3D ED models of Rb_2_Ru_2_O_7_·H_2_O (ICSD depositions: CSD Nos. 2514706 and 2464145, respectively), SEM, TEM, and PXRD with internal standard data (PDF)
