# Supplementary material for: Magnetism and 3D Electron Diffraction Solution of Hydrated Rubidium–Ruthenium Oxide Rb2Ru2O7·H2O
Source: Inorg Chem. 2026 Feb 18;65(8):4364–6. doi: 10.1021/acs.inorgchem.6c00345 (PMC12958284; doi:10.1021/acs.inorgchem.6c00345)
Supplement: Supplementary file 1 [file ic6c00345_si_001.pdf]

Supporting Information: Magnetism and 3D Electron Diffraction Solution of Hydrated Rubidium–Ruthenium Oxide $Rb_2Ru_2O_7{\cdot}H_2O$

Krystof Chrappova,[†] Jeremiah P. Tidey,[‡] Christopher Bell,[¶] and Simon R. Hall[*,†]

† *School of Chemistry, University of Bristol, Cantock's Close, Bristol BS8 1TS, U.K.*

‡*Department of Physics, University of Warwick, Coventry, CV4 7AL UK*

¶*H.H. Wills Physics Laboratory, University of Bristol, Tyndall Avenue, Bristol BS8 1TL, UK*

E-mail: simon.hall@bristol.ac.uk

This PDF contains synthetic procedure, crystallographic, magnetic property measurements, and electron microscopy details.

Synthetic Procedure

Rubidium carbonate (0.347g, 1.5 mmol) and ruthenium oxide (IV) (0.100g, 0.75 mmol) were ground together with a mortar and pestle until a homogeneous powder with uniform color was obtained. The decomposition temperature of rubidium carbonate is lower than the synthesis temperature. For this reason, an excess of rubidium carbonate was used in the synthesis, to account for loss through decomposition. Synthesis was carried out in an alumina crucible in a box furnace at 1000°C, for 4 hours at ramp rate 5°C min^{-1}. The product was as a dark brown powder obtained after air-cooling in the furnace.

Crystallographic Details

For the 3D ED experiment, the sample was dispersed dry as received onto a copper-supported holey amorphous carbon TEM grid and loaded at 100 K via a high-tilt Gatan Elsa 698 specimen holder into a Rigaku XtaLAB Synergy-ED electron diffractometer, operated at 200 kV and equipped with a Rigaku HyPix-ED hybrid pixel array area detector. Data were collected on various crystallites as single-rotation scans collecting 0.25 ° frames using CrysAlisPRO system (CCD 1.171.43.129a 64-bit (release 29-06-2024))[S1] using continuous rotation electron diffraction. A selected area aperture of 2 μm apparent diameter was used in the final dataset employed in the dynamical refinement, while the addition two contributing to the kinematic, SHELX-compatible dataset were collected with an aperture of 1 μm apparent diameter. Further experimental details are provided in Table S1. All single crystal component datasets were individually indexed and integrated, prior to the merging of three suitable datasets for scaling, using CrysAlisPRO (version 1.171.44.113a)[S1] ; no absorption corrections or outlier rejections were applied. The structure was solved using ShelXT[S2] and initially refined using Olex2.refine in the kinematic approximation, applying an extinction correction to broadly account for multiple scattering as implemented in Olex2 (version 1.5-ac7-016, compiled 2025.05.29 svn.r8cd99b3d for Rigaku Oxford Diffraction, GUI

svn.r7254)[S3,S4] and using published scattering factors.[S5] The resulting atomic coordinates were used as the starting point for a full dynamical refinement against the two highest quality data collections which were individually reduced and scaled without outlier rejection in CrysAlisPRO (version 1.171.44.128a).[S1] The dynamical reflection files were concurrently imported into JANA2020 (version 2.1),[S6] taking as reference unit cell parameters those for the highest quality component, and refinement was performed in the presence of ribbon models of thickness and anisotropic models of incoherent mosaicity for both datasets. All non-H atoms are refined anisotropically and the proton, earlier located in the residual difference map, was refined isotropically, all without the application of any restraints. We take as our final model to be the dynamically refined model, yet deposit CIFs for both outputs in the ICSD: deposition numbers CSD 2464145 and 2514706 for the SHELX-compatible kinematical (Table S1) and JANA2020 dynamical (Table S2) refinements, respectively. These contain complete experimental and refinement information along with appropriate structure factors and, in the former case, an embedded .RES file.

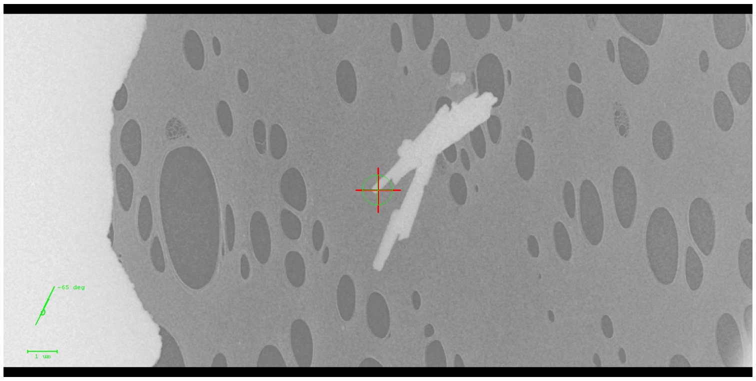

(a)

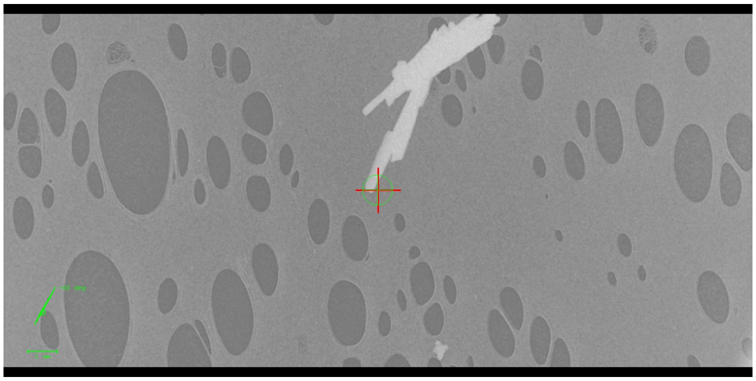

(b)

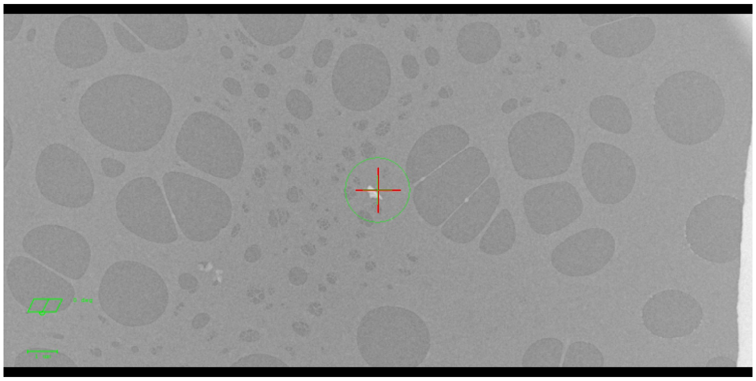

(c)

Figure S1: Images (a–c) correspond to the specific crystallites from which the data were collected. The 1 μm scale bar is shown bottom-left of each image, alongside the tilt angle at which the image is taken, while the green circle in the center represents the approximate location of the selected area aperture used in each experiment.

Table S1: Experimental details for the kinematic refinement against the merging of datasets collected on three distinct crystallites. The according CIF is deposited in the ICSD with Deposition Number CSD 2464145.

**Crystal data**	
Chemical formula	$Rb_2Ru_2O_7(H_2O)$
M_r	503.8
Crystal system, space group	Monoclinic, $C2/c$
Temperature / K	100(5)
a, b, c / Å	7.8575(18), 12.485(3), 8.3864(19)
α, β, γ / °	90, 94.10(2), 90
V / Å^3	820.6(3)
Z	4
Radiation type	Electron, $\lambda = 0.0251$ Å
**Data collection**	
Scan range / °	See `_diffrn_measurement_details` in CIFs for individual component angular ranges
Measured, independent, observed $[I \geq 2u(I)]$ reflections	5593, 999, 790
R_{int}	0.209
$(\sin\theta/\lambda)_{\text{max}}$ / Å^{-1}	0.674
**Refinement**	
R_1, wR_2 ($[F^2 > 2\sigma(F^2)]$)	0.1455, 0.2949
R_1, wR_2 (all)	0.1723, 0.3110
GoF (S) (incl.,excl. restraints)	1.0495, 1.0562
No. reflections	999
No. parameters	63
No. restraints	12
$\Delta\phi_{\text{max}}$, $\Delta\phi_{\text{min}}$ / eÅ^{-1}	1.69, −1.84

Table S2: Experimental details for the dynamical co-refinement against distinct datasets collected on two crystallites. The according CIF is deposited in the ICSD with Deposition Number CSD 2514706.

**Crystal data**	
Chemical formula	$Rb_2Ru_2O_7(H_2O)$
M_r	503.8
Crystal system, space group	Monoclinic, $C2/c$
Temperature / K	100(5)
a, b, c / Å	7.841(3), 12.500(3), 8.392(2)
α, β, γ / °	90, 93.57(4), 90
V / Å^3	820.9(4)
Z	4
Radiation type	Electron, λ = 0.0251 Å
**Data collection**	
Measured, observed $[I \geq 2u(I)]$ reflections (combined(component 1+2))	4241 (2236+2005), 2682 (1501+1181)
R_{int} (component 1; 2)	0.1514, 0.2044
$(\sin\theta/\lambda)_{\text{max}}$ integ. limit / Å^{-1}	0.674
**Refinement**	
$(\sin\theta/\lambda)_{\text{max}}$ data cutoff / Å^{-1}	0.70
Completeness at θ_{max}	0.95
g_{max} / Å^{-1}	1.6
Thickness	Ribbon
Mosaicity	Isotropic incoherent
R, wR, GoF, ($[I > 2\sigma(I)]$)	0.1049, 0.2501, 1.01
R, wR, GoF (all)	0.1315, 0.2753, 1.14
No. reflections, No. of which rejected ($\|F_o - F_c\| > 4\sigma(F_o)$)	4241, 12
No. parameters	118
No. restraints	0
$\Delta\phi_{\text{max}}$, $\Delta\phi_{\text{min}}$ / e Å^{-1}	1.36, −0.98

PXRD was measured on a Bruker D8 Advance diffractometer equipped with a PSD LynxEye detector using Cu–Kα radiation (λ = 1.542 Å). Rietveld refinements and crystalline fraction calculations were performed in Profex 5.5.0.[S7] The Rietveld refinement as described in the main text (Figure 2) was performed without relaxation of internal coordinates, only the unit cell (a, b, c, β), isotropic displacement parameter, sample displacement parameter, profile and preferred orientation were refined. The post refinement model gives a = 7.884(2) Å, b = 12.615(3) Å (fixed), c = 8.460(1) Å, β = 93.91(7)°.

For amorphous-phase quantification, Al_2O_3 (3.7 mg, 35.2 wt%) was mixed with the solid-state $Rb_2Ru_2O_7{\cdot}H_2O$ product (6.8 mg). The PXRD pattern of this mixture (Figure S2) was refined using the $Rb_2Ru_2O_7{\cdot}H_2O$and $\mathbf{Al_2O_3}$ (ICSD 130950) structural models, and the refined mass fractions from the Rietveld refinement were converted to the absolute mass fractions in the original sample using the internal standard method in Profex.[S7] This analysis indicates that 27 wt% of the solid state product is crystalline $Rb_2Ru_2O_7{\cdot}H_2O$.

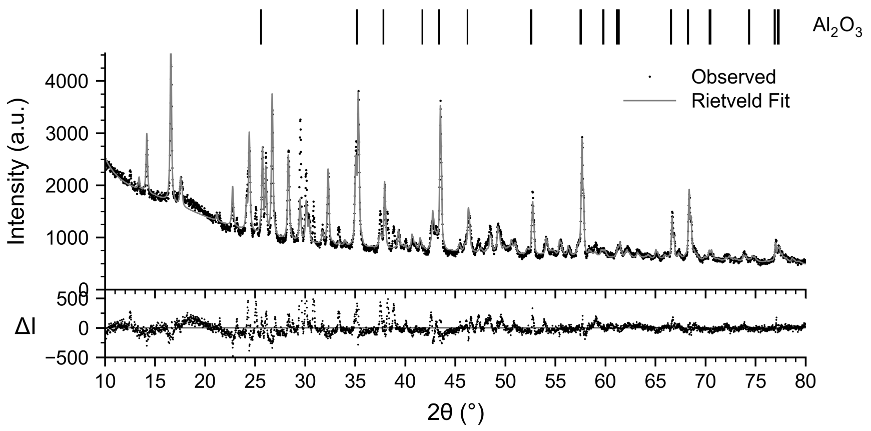

Figure S2: PXRD pattern of bulk $Rb_2Ru_2O_7{\cdot}H_2O$ with internal standard $\mathbf{Al_2O_3}$ Internal standard (ICSD 130950).

Magnetic property measurement

Experiments were carried out on a *Quantum Design MPMS3* magnetic property measurement system equipped with the AC and VSM options (MPMS3 Measurement Release 1.1.16,

Build 433; control software *MultiVu* v2.3.4.26). A 20 mg powder sample was wrapped in 4.4 mg of cling film, enclosed in gel capsules, and mounted in a straw holder.

DC mode The sample was cooled to 1.8 K in zero field, a 1 mT field was applied, and $M(T)$ was recorded while warming to 300 K at a rate of 1 K min^{-1}. Isothermal $M(H)$ loops were collected at 1.8 K (five quadrants, $0 \rightarrow 7$ T $\rightarrow$ -7 T $\rightarrow$ 7 T) and at 10, 20, 30, 40, 50, 60, 70, 100, 150, 200, 250, 300 K (four quadrants, ± 7 T). DC scans used a 30 mm scan length and 4 s scan time.

VSM mode. The 1 mT $M(T)$ warming measurement was repeated in VSM operation with a 5 mm drive amplitude and 2 s averaging time. Additional $M(H)$ loops at the same temperature set were measured with identical VSM settings; for comparison, DC scans were performed with a 10 mm scan length and 1 s scan time.

Scanning and Transmission Electron Microscopy

SEM analysis was performed on a JEOL JSM-IT300 system. The micrograph as shown in S3 revealed two morphologies: larger platelet-like structures, up to 12 μm in size, mostly located in the rubidium zone, and smaller clusters of particles present in the mixed metal regions. The elemental mapping micrographs in S4 show presence of both Rb and Ru and an overlap of the metals in the solid-state product.

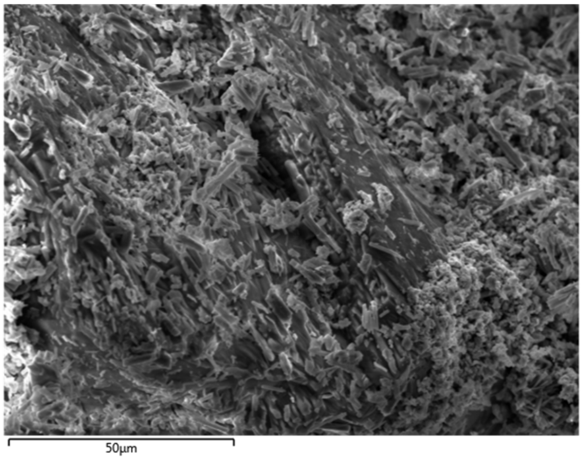

Figure S3: Scanning electron micrograph of solid state product of $Rb_2Ru_2O_7 \cdot H_2O$

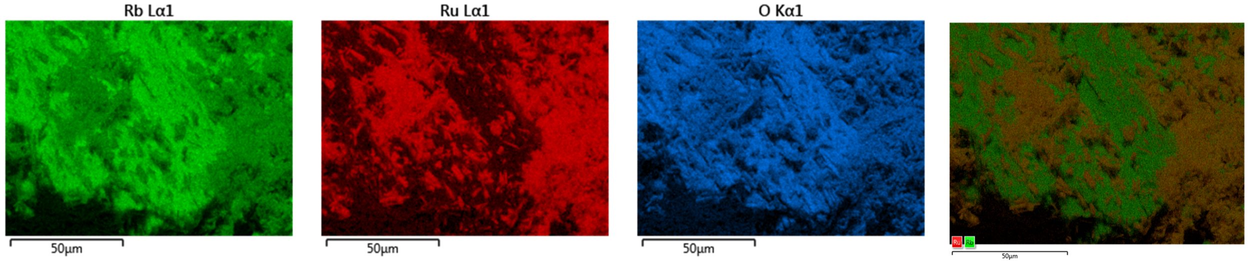

(a) (b) (c) (d)

Figure S4: Scanning elemental mapping micrographs of synthesis solid state product showing the distribution of (a) Rb (b) Ru (c) O (d) overlay (combined map) of Rb and Ru.

TEM analysis was performed on the JEOL JEM-1400 system reveals rod-like diffracting polycrystalline particles up to 6 μm in length.

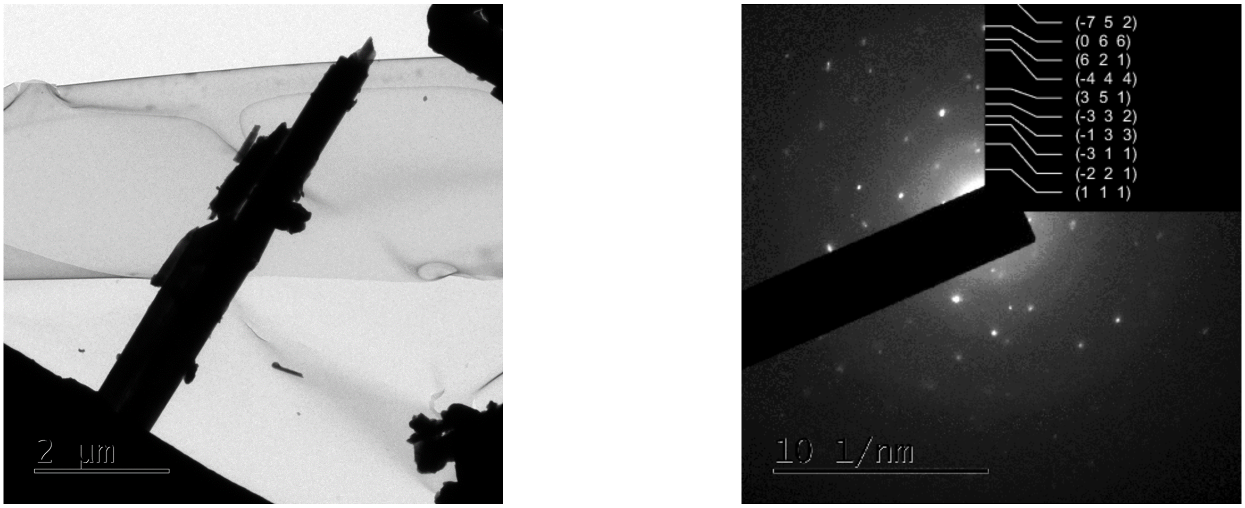

(a) (b)

Figure S5: TEM analysis of solid-state product where (a) shows the transmission electron micrograph of the solid state product crystal with a 2 μm at the bottom left of the image, and (b) shows the electron diffraction pattern obtained from the solid-state product synthesis.

The rings from the obtained diffraction micrograph were indexed to $Rb_2Ru_2O_7{\cdot}H_2O$(CSD 2514706) using CrysTBox ringGUI, and are listed in Table S3.[S8,S9]

Table S3: Ring identification from the diffraction pattern. Listed are theoretical and measured ring radii and corresponding d-spacings.

**Plane**	**Radius / nm^{-1}**		**d-spacing / nm**	
	**theor.**	**measured**	**theor.**	**measured**
(1 1 1)	1.972	1.936	0.507	0.516
(-2 2 1)	3.184	3.128	0.314	0.320
(-3 1 1)	4.024	4.022	0.249	0.249
(-1 3 3)	4.433	4.419	0.226	0.226
(-3 3 2)	5.002	4.990	0.200	0.200
(3 5 1)	5.718	5.685	0.175	0.176
(-4 4 4)	7.492	7.497	0.133	0.133
(6 2 1)	7.994	7.993	0.125	0.125
(0 6 6)	8.623	8.614	0.116	0.116
(-7 5 2)	9.952	9.955	0.100	0.100

References

(S1) Rigaku Oxford Diffraction, CrysAlis PRO. **2025**,

(S2) Sheldrick, G. M. SHELXT– Integrated space-group and crystal-structure determination. *Acta Crystallographica Section A Foundations and Advances* **2015**, *71*, 3–8.

(S3) Bourhis, L. J.; Dolomanov, O. V.; Gildea, R. J.; Howard, J. A. K.; Puschmann, H. The anatomy of a comprehensive constrained, restrained refinement program for the modern computing environment –Olex2 dissected. *Acta Crystallographica Section A Foundations and Advances* **2015**, *71*, 59–75.

(S4) Dolomanov, O. V.; Bourhis, L. J.; Gildea, R. J.; Howard, J. A. K.; Puschmann, H. OLEX2: a complete structure solution, refinement and analysis program. *Journal of Applied Crystallography* **2009**, *42*, 339–341.

(S5) Saha, A.; Nia, S. S.; Rodríguez, J. A. Electron Diffraction of 3D Molecular Crystals. *Chemical Reviews* **2022**, *122*, 13883–13914.

(S6) Petříček, V.; Palatinus, L.; Plášil, J.; Dušek, M. Jana2020 – a new version of the

crystallographic computing system Jana. *Zeitschrift für Kristallographie - Crystalline Materials* **2023**, *238*, 271–282.

(S7) Doebelin, N.; Kleeberg, R. Profex: a graphical user interface for the Rietveld refinement program BGMN. *Journal of Applied Crystallography* **2015**, *48*, 1573–1580.

(S8) Klinger, M.; Jäger, A. Crystallographic Tool Box (CrysTBox): automated tools for transmission electron microscopists and crystallographers. *Journal of Applied Crystallography* **2015**, *48*.

(S9) Klinger, M. CrysTBox - Crystallographic Toolbox. 2015; `http://www.fzu.cz/~klinger/crystbox.pdf`.
